# Simulation and Training in Robot-Assisted Urological Surgery: From Model to Patient

**DOI:** 10.3390/jcm13061590

**Published:** 2024-03-10

**Authors:** Flavia Proietti, Rocco Simone Flammia, Leslie Claire Licari, Eugenio Bologna, Umberto Anceschi, Maria Consiglia Ferriero, Gabriele Tuderti, Riccardo Mastroianni, Aldo Brassetti, Giuseppe Simone, Costantino Leonardo

**Affiliations:** 1 Department of Urology, IRCCS Regina Elena National Cancer Institute, Via Elio Chianesi 53, 00144 Rome, Italy; 2 Department of Maternal and Child Health and Urological Sciences, Policlinico Umberto I, “Sapienza” University of Rome, Viale del Policlinico 155, 00161 Rome, Italy

**Keywords:** simulation, training, virtual reality, dual console, robot-assisted surgery, robotic curricula, urology

## Abstract

(1) Background: Simulation-based training has revolutionized surgical education, providing a solution to the changing demands of surgical training and performance. The increasing demand for standardized training in robotic surgery has accelerated the adoption of simulation-based training as a necessary component of modern surgical education. This study examines the existing literature on training approaches employed in robot-assisted urological surgery; (2) Methods: The authors conducted a standardized search of online databases. Upon collecting the articles, the authors assessed their relevance and content before proceeding with the drafting of the text; (3) Results: The use of simulators is supported by convincing evidence that shows an advantage in the acquisition of robotic skills. Urological societies have created detailed training programs for robotic surgery that guide beginners through the entire process of skill acquisition; (4) Conclusions: The future landscape for robotic urology training is likely to involve organized, obligatory, and centralized training, which may be overseen by urologic associations.

## 1. Introduction

The training method “See one, Do one, Teach one”, based on the role of the mentor, has been the fundamental principle of medical education, particularly in surgery, for decades. This quote dates back to the late 19th century, attributed to Dr. William Halstead, the founder of Johns Hopkins University School of Surgery. However, in recent years, this principle has been challenged due to the increasing attention towards surgical outcomes and medico-legal aspects. In fact, numerous studies have highlighted the importance of surgical skills in determining patient outcomes, including mortality rates, complication rates, operation duration, re-operation rates, and readmission rates. It is interesting to note that surgical skills can account for up to 25% of the variation in patient outcomes [1]. These aspects have led to a significant revolution in surgical training. This revolution is attributable to the introduction of increasingly sophisticated and realistic simulation systems, which have become the core of advanced education programs. Simulation systems have been imported from other high-risk professions, such as aviation. The first simulation system was a flight simulator, known as the “Link Trainer”, created by Edwin A. Link in 1929 [2], to teach new pilots. The first application in the medical field was published by P. Safar in 1958 [3], and the first full-sized training mannequin, Resusci-Anne, was created soon after. The first simulator for surgical training was developed in 1989 by NASA members Rosen and Delp and consisted of a representation of a lower limb designed to practice tendon transplants for reconstructive surgery [4]. Since then, increasingly advanced simulation systems have been developed. In addition, the growing popularity of minimally invasive surgeries like endoscopy and laparoscopy, particularly in urology, has led to an increased demand for simulation systems to help train and prepare surgeons for these procedures. In the last two decades, there has been a tenfold to fortyfold increase in the number of surgeries performed using robot-assisted technology, compared to traditional laparoscopy [5]. Therefore, there is a growing demand for urologists capable of using robotic technology and the need to introduce a standardized training curriculum. In this review, we aim to examine the current status of training in robotic surgery, which encompasses different methods and tools. We also discuss the gradual approach to training new surgeons in robotic surgery techniques, with a focus on the use of structured training programs.

## 2. Materials and Methods

To provide a comprehensive overview of simulation models and training programs, we conducted a narrative review. The PICO framework was employed to facilitate the search process with a focus on the following specific parameters:

- Population: Urologists;
- Intervention: Education and training;
- Comparison: Simulation systems and training programs versus mentor-based education;
- Outcome: Acquired skills and surgical outcomes.

A bibliographic search of the PubMed and SCOPUS databases was performed to identify all articles concerning a training and simulation system. A combination of the following keywords was used for the search: “urology”, “simulation”, “robotic curricula”, “surgical education”, “surgical training”, “advanced training”, “virtual reality”, and “dual console”. The latest search was returned and finalized in December 2023. All studies on the topic of interest have been included, regardless of the type of study. We manually searched through the references cited in the studies included in the screening process, as well as relevant review articles, in order to identify any additional eligible studies. Articles that were either not available in English or did not have the full text available were excluded. Manuscripts regarding training in pediatric urology were also excluded. We considered articles published in the last 20 years, with a particular focus on the last 5 years. All results were first screened by title and abstract from two independent authors (E.B. and L.C.L). All of the remaining results and any studies considered “dubious” were examined in full. The identification, screening, and selection of the scientific articles to be included in our review were supervised by two experienced supervisors (C.L. and G.S.). The results of our search were reported according to the PRISMA statement checklist [6].

## 3. Results

**Figure 1:**
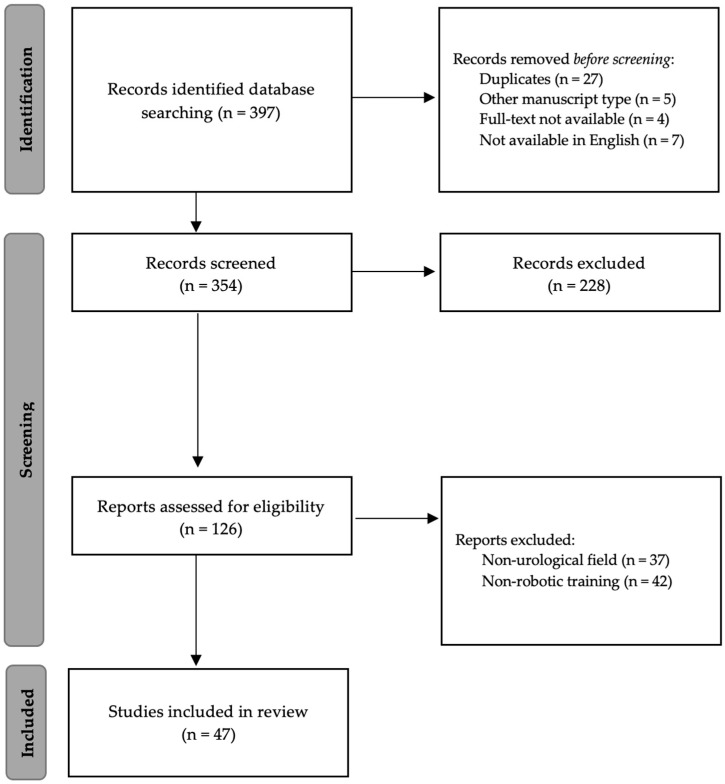
Flow diagram of the selection process regarding robotic training (PRISMA guidelines).

The literature search yielded 397 papers. Through a manual process, 174 records were removed. After screening the titles and abstracts of the remaining 354 references, we excluded 228 studies. Thereafter, we assessed the full texts of the remaining 126 studies for eligibility, and 47 were accepted. The flow diagram (Figure 1) provides a graphical representation of the literature search and screening process.

### 3.1. Simulation Training

#### 3.1.1. Rational of the Simulation System

In applied sciences, “simulation” refers to a real-life-based model that reproduces situations for training purposes. The goal of simulation systems is to facilitate learning in safe and low-stress conditions, allowing for standardization, completeness, and personalization of training while objectively assessing performance.

The use of a simulation system for surgical training should involve the acquisition of both technical and non-technical skills. As far as technical skills are concerned, traditional surgical training has relied on the knowledge of anatomy and surgical techniques, gradually applied on a real patient under the supervision of an experienced surgeon. However, it has been demonstrated that the development of technical ability using these methods is inefficient, time consuming, and costly [7]. On the other hand, in recent decades several randomized studies have reported the usefulness of undergoing surgical training on simulation systems, demonstrating that this approach results in better performance in the operating room [8,9]. Non-technical skills, on the other hand, include skills such as situational awareness, decision making, leadership, and teamwork. These abilities, improved through simulation, are the main determinants in crisis management, as demonstrated by studies conducted in the field of anesthesia and surgery [10,11]. It is crucial to include the training of non-technical skills in any simulation-based training program. These skills can be honed through hands-on experience and specialized training in crisis management (known as “Crisis Resource Management” (CRM)).

Finally, in addition to the technical and non-technical aspects, the psychological impact and the effect of stress have been considered. In 1908, Yerkes and Dodson demonstrated that stress, as well as learning efficiency and performance, follow the inverted U-shaped curve. However, there is a performance drop when stress becomes too high. It has been shown that the use of simulators can reduce the level of stress of trainee surgeons. On the other hand, a moderate level of stress should be added to the simulation system, as this is known to be the optimal context for psychological learning [12].

#### 3.1.2. Ideal Features of Medical Simulator

A simulator should meet the ideal criteria as follows:

- Fidelity: It concerns the simulator’s ability to accurately replicate reality, both in terms of appearance (e.g., surgical field) and functionality (e.g., wrist movement).
- Replicability: It refers to the ability to replicate or repeat the same setting. It involves conducting the exercise, using the same methods and procedures, and obtaining identical results. Replicability is an important aspect of training because it allows for the standardization of a task and, therefore, the possibility of measuring and quantifying progress. It also ensures the exportability of a training protocol.
- Cost: The cost of simulators represents a critical feature. In fact, to obtain the aforementioned characteristics, especially if considering virtual reality, a team of experts is necessary to design and produce a simulator, including engineers and programmers; this commitment results in an increase in high costs. Wet labs have also always been considered high cost both for the acquisition and management of biological models.
- Portability: Portability refers to the ability of a device, software, or technology to be easily transported or transferred between different systems or platforms, without affecting its functionality or performance.

In any case, it is necessary to consider that a single device may not necessarily fulfill all the possible skills to acquire, and that training should be the result of the subsequential skills acquired in different programs and contexts.

Moreover, there are limitations to using simulators for training in surgery. There is still disagreement on which tasks and exercises are applicable in real-world scenarios. Measuring competence based on time-to-perform procedures is not always effective in clinical settings due to various factors influencing performance. Additionally, cognitive and human factors play a significant role in displaying skill during surgery, making it challenging to predict the validity of simulation training. Furthermore, variations in anatomy and pathology contribute to the challenging nature of surgical training, and simulated tasks can only provide the initial foundation for building clinical expertise [13].

#### 3.1.3. Simulation Systems

Simulation-based training is becoming more widely acknowledged as a beneficial addition to urology and other skilled disciplines. It allows trainees to hone basic skills in a secure setting, ensuring patient safety and aiding in the early stages of learning. There are multiple methods available, such as virtual reality (VR) and bench-top simulators, along with animal and cadaver models, each with its own pros and cons [14]. 

Dry labs—Dry labs use nonhuman and nonanimal “covered boxes” (such as the pelvic trainer) for training in robotic procedures. They help develop hand–eye coordination and allow the surgeon to gain familiarity with the specific video endoscopic field. Endotrainer boxes are commonly employed to develop skills such as ambidexterity, pattern cutting, needle positioning, suture, and knot tying. Some surgical steps are available, such as vesico-urethral anastomosis. This method is a cost-effective means of teaching basic psychomotor abilities and ensures standardized training across the globe. However, it offers the least surgical realism among simulation techniques, resulting in decreasing benefits for each skill acquired and limitations in evaluation beyond basic measurements like time to completion or error prevention [15]. Conversely, Raison et al. reported that, for more advanced skill training, dry-lab training was found to be superior to VR simulation [16]. Moreover, there is not much proof to suggest that VR simulators enhance dry-lab skills; junior and experienced surgeons did not showcase any advancement in their dry-lab skills after participating in a virtual reality simulation [17].

Wet labs—In wet lab simulations, organic tissues are utilized to create a more authentic training environment for practitioners to develop their skills in handling tissue and understanding its response to instruments and diathermy. This form of simulation offers tactile feedback that can be applied in an operating theater setting. Examples of wet lab training models include synthetic models, animal, and cadaveric labs. Cadaver labs have the advantage of providing training on real human anatomy (high fidelity); however, the simulation deviates from reality due to the absence of respiratory movements, bleeding, and energy-induced muscle contractions. Moreover, training sessions are difficult to organize, due to legislative issues, the availability of cadavers, and requiring dedicated surgical equipment. Animal laboratories (e.g., pigs) have the advantage of creating an environment that is as close to reality as possible but require anesthesia support and dedicated surgical equipment. In certain instances, the models do not accurately mimic the human process, such as in the case of porcine models used for radical prostatectomy. Additionally, it has raised ethical concerns on several occasions. 

Both cadaver and animal laboratories are reserved for a few reference centers with long waiting lists. Moreover, they also have the disadvantage of high costs. 

They allow the development of skills such as patient and port positioning, appropriate use of energy, dissection, up to the possibility of performing entire surgical procedures [18]. On the other hand, Lovegrove et al. reported that there is little to no compelling evidence that a particular wet-lab exposure leads to enhanced outcomes [19].

Synthetic organ models—Efforts have been ongoing for some time to replace live animal and cadaver surgery with various physical models being developed, including those made from artificial materials like sponges, tubes, 3D-printed models, and tissue-based models [20,21]. Their limitations include a lack of anatomical precision, limited haptic feedback, inadequate color accuracy and similarity, lack of electrosurgical functionality, and absence of bleeding simulation [22]. A 3D-printed model allows for the production of personalized models representing a patient’s anatomy or medical condition in difficult cases [23], as opposed to the mass production of identical parts typically created through subtractive manufacturing [24]. Simulation-based education employing 3D models is recognized for robot-assisted radical prostatectomy, partial nephrectomy, pyeloplasty, and kidney transplant [24,25]. At present, the only method to simulate tissue texture that closely resembles real human organs is through injection casting. This technique involves using 3D printing to replicate the specific morphology of organs, with the ability to accurately mimic the physical properties such as tissue textures, cauterization, suturing, and dissection planes based on the type of polymer used. Ghazi validated the efficiency of a simulation platform for robot-assisted partial nephrectomy training that utilized perfused PVA kidney phantoms. These phantoms were made using 3D-printed molds based on a patient with a 4.2-cm exophytic renal tumor [26]. Other models have been created, including a model of nerve-sparing robot-assisted radical prostatectomy using a wire sensor to measure tension that is integrated into the neurovascular bundle [27,28].

Virtual reality—Virtual reality (VR) is technology that enables individuals to interact with a real-time computer-generated three-dimensional reality, using their natural senses and abilities. The interaction is recorded and interpreted by the computer, which is able to generate a coherent response. The availability and immersive nature of VR surgical training is growing, enabling trainees to enhance their essential skills by repeating tasks in a controlled setting [29,30]. Additionally, it provides a report of the trainee’s performance, which is useful for creating a tailored training program. This results in shorter surgery durations, decreased occurrence of complications, and better overall results [31]. However, it has the disadvantages of having a high initial cost [22], requiring maintenance, and having poor or no haptic feedback [14]. 

This technology was initially used in space flight and aviation training, and only later was applied in the medical field to train surgeons. Video-assisted procedures, particularly robot-assisted surgery, are particularly well suited to the development of virtual reality models, as the movements of robotic masters can be broken down into simple movements (such as rotational or linear motion), easily interpretable by a computer.

The most commonly used simulator is the da Vinci TM Skills Simulator (dVSS) (Instuitive Surgical, Sunnyvale, CA, USA), which is compatible with Si and Xi systems and features 30 exercises to assess nine robotic skills. Performance is graded based on feedback provided on overall score and proficiency in time management and movement. Deductions are made for penalties such as collisions, excessive force, incorrect targeting, and movements outside of the user’s line of sight. Users may record and track their progress, while a supervisor can monitor their results. Additional complex programs are available for specific procedures, such as radical prostatectomy, with detailed reports provided at the end of each module. Other standalone systems are also available, such as the Mimic dV-Trainer (MdVT) (Mimic, Seattle, WA, USA), which assigns weightings to metrics such as bimanual dexterity, task time, safety in the operative field, and number of critical errors to provide an overall score comparable to the da Vinci TM. The RobotiX Mentor (RM) (CD Systems, Simbionix Products, Cleveland, OH, USA) represents the latest addition to the market in 2016; however, the dVSS scored highest in face and content validity compared to the RM and MdVT [32]. Other VR simulation systems include the ProMIS simulator (ProMIS) (Haptica, Ireland), the Surgical Education Platform (SimSurgery, Oslo, Norway), and the Robotic Surgical Simulator (RoSS) (Simulated Surgical Systems, San Jose, CA, USA) [22,33]. These platforms offer a variation of the daVinci robotic console, with the dVSS being the only exception that connects to the surgical console. They provide a library of exercises focusing on basic skills such as camera control, clutching, endowrist manipulation, and fourth arm use. While they offer residents the opportunity for unlimited repetitive training, they come with a hefty price tag of over $100,000 [33]. New robotic platforms are spreading, and with them, specific simulation systems have been developed. Among these, the Hugo RAS^TM^ simulator (Medtronic, Minneapolis, MN, USA) has shown to be effective in improving surgical skills [34].

In recent years, artificial intelligence (AI) has been introduced and integrated into virtual reality-based simulation systems. AI focuses on developing an independent computer system that can perform tasks like humans. It does this by using complex mathematical models that mimic the structure and function of the human brain [35]. These models are composed of non-linear systems, allowing the AI to learn and make decisions in a flexible and intelligent manner. The main applications involve performance evaluation and the implementation of training model fidelity [36].

Augmented reality—Augmented reality (AR) has recently been introduced in the medical field [37]. AR offers a different experience compared to VR, as it enables haptic feedback and interaction within a virtual setting. Unlike VR’s fully immersive digital environment, AR superimposes digital information onto the real world, thus merging the advantages of traditional tactile training methods and VR. For this reason, it is increasingly being used in robotic training as it provides a more interactive, engaging, and effective way of learning and training. The main advantages are represented by (1) Enhanced visualization: AR provides a 3D visualization of robotic systems and components, making it easier for trainees to understand their structure, operation, and interaction; (2) Real-time feedback: AR can provide real-time feedback to trainees during robotic training exercises; (3) Interactive Learning: AR provides an interactive learning experience, allowing trainees to manipulate and interact with virtual robotic systems; (4) Safe training environment: Trainees can practice working with virtual robotic systems without the risk of damaging expensive equipment or injuring themselves; (5) Customizable training: AR allows for customizable training programs that can be tailored to the individual needs and learning styles of trainees. This personalized approach can help to improve learning outcomes and reduce training time; (6) Cost effective: despite the high cost of the software development, AR can reduce the cost of robotic training by eliminating the need for physical robotic systems and reducing the need for travel and accommodations. AR can also reduce the cost of maintenance and repairs associated with physical training systems; (7) Remote training: AR allows for remote training, enabling trainees from different locations to participate in training programs simultaneously. This can help to reduce travel time and costs, making training more accessible and convenient.

As AR technology continues to evolve, its role in robotic training is likely to become even more significant [38]. Even in this type of technology, the integration of artificial intelligence is possible: software’s that are increasingly complex and accurate are being researched [39].

### 3.2. Training Programs

A training program, as such, should be organized according to a sequence; participants should acquire basic skills before moving on to intermediate ones, then advanced ones, and finally training on entire procedures. In this way, participants will be able to develop a solid foundation of knowledge and skills before moving on to more complex tasks. With this approach, the training path will flow naturally from simple to complex, providing participants with a solid base and gradually increasing their understanding and competence.

Hands-on Training—Hands-on Training (HoT) involves acquiring practical skills where one is able to directly use surgical instruments and practice the skills being learned. The term “HoT” is generally used to describe brief training sessions typically lasting one hour. Each training station is equipped with a standardized set of exercises and instruments that follow a specific training protocol. Given the limited time, the instructor adheres to a strict schedule in order to allocate sufficient time to each task.

Boot Camps—A boot camp is a concentrated program geared towards improving education, orientation, and readiness for those starting a new clinical position. This is accomplished by employing various teaching techniques that focus on deliberate practice and providing developmental guidance. Typically, boot camps span over a period of 3–5 days and are conducted in various small groups. Participants undergo multiple HoT sessions, each with a clear objective to reach at the end of the program.

The European School of Urology (ESU) has developed the Standardisation in Surgical Education (SISE) program. The SISE program consists of a sequence of standardized and validated training modules, which includes a laparoscopic urology training (LUS) curriculum. The initial phase of the SISE program involves participation in the ESU Urology Boot Camp (ESU UBC), where first-year residents undergo a standardized course. This course involves a full day of immersive practical training, aimed at equipping urology residents with the fundamental technical skills required for common urological procedures. The primary goal is to ensure that residents attain proficiency in these essential skills before they start their clinical practice with patients.

Robotic training curricula—In the past years, the development of expert robotic skills was a non-standardized and lengthy process. Nowadays, trainees and surgeons in the medical field are required to have a comprehensive education in robotic training, which means they need to follow a structured step-by-step approach that covers all aspects of the learning process, starting from beginner level and leading to advanced proficiency. This process should include a pre-console training and a console training. The first step, which can be applicable to all surgical procedures, aims to acquire skills such as patient positioning, port placement, camera and instrument manipulation, management of pneumoperitoneum, and spatial awareness. It has been proven that the development of these skills is associated with a reduction in execution time and a lower error rate in the operating room [40]. When robotic training is considered, an extensive knowledge of the system components (software, robotic arms, column and console settings, etc.) is necessary; in fact, errors in managing the machine could be dangerous for the patient or cause expensive damages. This step should also include patient-side pre-console training, enhancing console performance and troubleshooting capabilities during surgery. However, the learning curve for this training has not been defined, and there is a lack of guidance on the number of cases that trainees should assist in, in order to gain proficiency. The console-side training represents the second step of the process and requires procedure-specific training. The training is composed of two phases: a pre-clinical stage and a clinical stage. To bridge the gap between these two stages, simulation training is utilized. This training encompasses dry lab, wet lab, and virtual reality exercises, all of which serve to equip trainees with the necessary skills to perform live procedures effectively [41]. In the operating theatre, the training process initiates with trainees observing and then advances to executing simpler tasks under the guidance of an experienced surgeon. As trainees improve their skills, the role of the supervisor transitions from being a preceptor (offering assistance as necessary) to a proctor (supervising while enabling trainees to take charge) so that trainees can perform with supervision. The trainee performs steps of increasing difficulty until executing the entire procedure.

By following the aforementioned steps, several robotic training curricula have been developed [42]. Among the most well-known are the British Association of Urological Surgeons (BAUS) curriculum and the European Association of Urology (EAU) Robotic Urology Section (ERUS) curriculum.

- The EAU/ERUS curriculum—In 2015, the EAU/ERUS board published the first validated robotic training program for robot-assisted radical prostatectomy (RARP) [43]. Subsequently, Larcher et al. proposed and validated a similar program for robot-assisted partial nephrectomy (RAPN) [44]. Finally, a structured training curriculum for robot-assisted radical cystectomy (RARC) with intracorporeal ileal conduit in male patients was recently defined through a Delphi consensus study [45]. All of these training programs follow the same training structure, which consists of theoretical training, preclinical simulation-based training, clinical modular training, and a final evaluation.
  Robot-assisted radical prostatectomy curriculum has been described as follows:
  1. Theoretical training—It includes e-learning and case observation.
  2. Preclinical simulation-based training—an intensive week of structured, simulation-based training with virtual reality synthetic, animal, and cadaveric platforms.
  3. Clinical modular training—Operative training using a modular approach dissects complex procedures into smaller steps, which are sequenced in order of execution. However, trainees do not have to follow the chronological order of the steps but can progress to more advanced stages as their skill level improves. The premise is that these stages require varying levels of expertise, and the program provides a systematic exposure to increasingly intricate stages. The EAU/ERUS educational committee has established a structured modular training framework for robotic-assisted radical prostatectomy that mandates a specific frequency of performing each step (Table 1).

EAU/ERUS has suggested a comparable plan for carrying out RAPN and RARC [44,45]. This includes determining the required number of repetitions for each step and determining an agreed-upon level of complexity for each step through consultation with an expert panel. After finishing the modular training, the surgical mentors used the GEARS score [46] to evaluate the RARP procedural capabilities of the participants. Additionally, they assessed the quality of surgical skills for every step of the RARP procedure by using a scoring scale of 1 to 5 specific to the RARP procedure, where 3 indicated acceptable performance. 

Final evaluation—The final RARP procedures performed by each fellow were evaluated with a linear scoring criterion for every procedural step by blinded, skilled robotic surgeons to determine if the candidate had achieved satisfactory levels in executing each step. 

The program lasts for 180 days.

- The BAUS curriculum—A curriculum document for modular training, encompassing robotic radical prostatectomy, pyeloplasty, partial nephrectomy, and cystectomy, has been proposed by the BAUS. This training program, similar to the EAU/ERUS program, consists of five steps: e-learning, observation of procedures, simulation-based training, a mentorship/fellowship period, and independent surgery.
- Novel robotic systems [47] are now introducing dedicated training programs as well. For instance, Medtronic’s Hugo Ascend training pathway offers both online and in-person training, allowing trainees to practice in a virtual environment and progress from pre-console training to preceptored surgical cases. The new platforms also include the Versius and the Avatera Robotic System, for which specific training programs are being developed [48,49,50].

### 3.3. Dual Console 

The da Vinci Surgical System’s “dual console” configuration has proven to be a highly effective solution for robotic surgical training limitations. It enables the supervising surgeon and their trainee to operate from separate master and secondary consoles. Despite the higher cost, dual console robotic platforms can greatly benefit surgical training. They enable trainees to observe procedures from an expert’s viewpoint and facilitate smooth collaboration and supervision. Moreover, the dual console allows the supervising surgeon to immediately take control of the robotic instruments. Research indicates that compared to single console procedures, the use of a dual-console system could potentially enhance the outcomes during the perioperative and intraoperative phases while teaching resident surgeons to perform robot-assisted radical prostatectomy [51]. As per their findings, using a dual-console system could be a better option in aspects of robotic surgical education compared to a single-console system because it may provide a safer and more effective means of training.

### 3.4. Comparison of Robot-Assisted Surgery Simulators

As previously mentioned, simulation-based training is increasingly being recognized as a valuable addition to training in urology and other skilled disciplines. It allows trainees to practice essential skills in a safe and forgiving environment, ensuring patient safety and facilitating progress along the initial phase of the learning process. Different modalities, such as virtual reality and bench-top simulators, along with animal and cadaveric models, offer their own sets of benefits and drawbacks. Bench-top simulators are universally the most widespread and cost-effective model. The possibility of being built at home [52] with easily available materials is not negligible. However, they have the disadvantage of not being very faithful to reality in terms of haptic feedback and the properties of the materials used. Simulation systems that use VR, 3D models, or animals/cadavers certainly have a higher cost [53]. However, they present, for different reasons, a greater fidelity to reality. Training on cadavers, in fact, has a very high anatomical correspondence, but does not have the physiological properties of the human body (respiratory excursions, circulation, peristalsis, etc.). These latter properties are found instead in live animal models, which however present variable anatomical differences, depending on the animal used (pig, chicken, etc.) [54]. Finally, virtual reality allows for recording and evaluating results, provides high-quality images but has high initial and maintenance costs, and lacks haptic feedback (Table 2) [14].

### 3.5. Future Directions

With growing evidence supporting the effectiveness of simulation in enhancing surgical skills, the focus now shifts towards maximizing its utilization rather than questioning its efficacy. Different efforts are underway by international associations to improve training programs, including various types of education and combining different simulation modalities [43,44,55]. The main issue continues to be the limited availability of training centers and, therefore, access to programs. 

## 4. Conclusions

Training programs focused on robotics and related skills are gaining popularity and are being refined to be more structured and organized. Professional organizations recognize the importance of standardized curricula for these programs. Various curricula are currently under development, and additional work is necessary to validate and implement them before standardized robotic training can become more widespread. Collaboration among professional societies, accredited training centers, and institutions is essential for the coordinated development and implementation of these curricula, leading to a consensus on standardized education and ultimately benefiting all involved. Augmented reality, virtual reality, and 3D-printed synthetic models are gaining popularity due to the absence of management and ethical issues, despite the costs being a significant aspect. Complete virtual operating rooms will eventually facilitate comprehensive surgical training, including technical and team skills, optimizing the learning environment for residents and practicing urologists interested in robotic surgery.

## Figures and Tables

**Table 1:** Modular scheme for training in robot-assisted radical prostatectomy proposed by EAU/ERUS.

Robot-Assisted Radical Prostatectomy Steps	Repetition Required
Bladder detachment	20
Endopelvic fascia incision	15
Bladder neck incision	15
Section of vas deferens and preparation of seminal vesicles	15
Dissection of the posterior plane	10
Dissection of the prostatic pedicle	10
Dissection of neurovascular bundles	5
Ligation of Santorini plexus	10
Apical dissection	5

**Table 2:** Available simulation models: pros and cons [14,52].

Model	Pros	Cons
Bench-top	Large availability	Low anatomic fidelity
	Lower cost	Different haptic feedback
	Portable/Reusable	Need of true operative equipment
	No ethical issue	Only specific task simulation
Animal	Complete procedure simulation	High cost
	Comparable anatomy	Ethical considerations
	Same tissue properties	Limited reusability
		Animal laboratory required
Cadaver	Complete procedure simulation	High cost
	Same anatomy	Different tissue properties
		Nonreusable
		Low availability
		Specific laboratory setup required
Virtual Reality	Reusable	High initial cost
	Performance data capture and feedback	Maintenance
	Specific laboratory setup not required	Low haptic feedback
	High fidelity *	Low availability
		Low fidelity *

* Fidelity depends on the design.

## Data Availability

No new data were created during this study.
